# A protocol for a single-center, single-blinded randomized-controlled trial investigating volar plating versus conservative treatment of unstable distal radius fractures in patients older than 65 years

**DOI:** 10.1186/s12891-019-2677-y

**Published:** 2019-06-29

**Authors:** Jonas Pedersen, Simon Oksbjerre Mortensen, Jan Duedal Rölfing, Rikke Thorninger

**Affiliations:** 1 Department of Orthopedics, Regional Hospital Randers, Skovlyvej 15, DK-8930 Randers, Denmark; 2 Department of Orthopedics, Aarhus University Hospital, Aarhus, Denmark; 3 Institute of Clinical Medicine, Aarhus University, Aarhus, Denmark

**Keywords:** Distal radius fractures, Volar plating, Conservative, Complications, Functional outcome, Elderly

## Abstract

**Background:**

Distal radius fractures (DRF) are very common in elderly patients, who present at the Emergency Department. Surgical treatment with open reduction and internal fixation using volar locking plates is widely prevalent despite the lack of evidence proving its superiority to conservative treatment with closed reduction and plaster immobilization. The purpose of this study is to investigate whether conservative treatment is superior to volar plating in terms of number of complications and results in a comparable or superior functional outcome in patients ≥65 years.

**Methods:**

In this single-center, single-blinded randomized-controlled trial, patients ≥65 years with distal radius fractures will be invited to participate. A total of 50 patients per treatment arm is required to provide 80% statistical power at a 5% alpha level assuming a difference of 20% in complication rate between operatively and conservatively treated patients. Primary outcome measures will be complication rate, Quick DASH score (Quick Disabilities of the Arm, Shoulder and Hand), PRWE (Patient rated Wrist evaluation), and range of motion of the wrist. Secondary outcome measures will be grip strength, pinch gauge, pain, use of pain medication EQ5D score (European Quality of life – 5 dimensions), standardized radiographs. One year of follow-up is planned with data collection at the day of injury, after 2 weeks, after 5 weeks, after 6 months, and after 12 months. An intention-totreat and per-protocol analysis will be performed.

**Discussion:**

This prospective trial helps to clarify the best treatment strategy for displaced DRF patients ≥65 years.

**Trial registration:**

This trial is approved by the Danish Scientific Ethical Committee (ID: 1–10–72-420-17) and registered at Clinicaltrials.gov (Trial registration number NCT03716661).

## Background

Distal radius fractures (DRF) account for 18% of all fractures in the elderly ≥65 years of age [1]. The estimated lifetime risk for DRF is 15% for females and 2% for males [2]. The incidence rate is 190–200 per 100,000 person-years [3]. DRF is associated with osteoporosis, hence the age-related incidence rate increases almost 3-fold from the age of 60 to 99 in women [1, 4, 5]. In Europe, the proportion of the elderly population is estimated to increase by 56% in men and by 41% in women until 2035 [6, 7]. Consequently, the need to clarify the best treatment strategy for DRF in elderly is evident.

In recent years, there has been a trend to treat DRF patients that require surgical treatment with an open reduction and internal fixation (ORIF) using a volar locking plate. The Danish Health Authority stipulates in the National Clinical Guidelines (NCG) regarding the treatment of low-energy DRF [8] to volar plate fractures that fulfill the following radiologic criteria after attempted closed reduction:

- > 10° dorsal tilt of the radius in relation to perpendicular to the longitudinal axis of radius
- > 2 mm articular step-off
- > 2 mm ulnar variance
- incongruence of the distal radioulnar joint
- substantial dorsal comminution indicating gross instability

If one or more of these criteria are met, ORIF most often utilizing a volar locking plate is advised regardless of the patient’s age. The guideline also highlights that conservative management should be considered in patients with low functional demands.

Notably, the guideline does not include recommendations for high-energy, open fractures nor grossly instable fractures: volarly displaced (Smith), radial styloid (Cheuffeur) or articular rim (Barton) fractures. However, most of these fractures are also treated with volar locking plates in Denmark.

The radiological NCG criteria rely on clinical observations only and have not been systematically evaluated prospectively. Furthermore, the reliability of the radiological criteria has been questioned [9]. Therefore, The Danish Health Authority evaluates that the recommendations for treating DRF primarily are based on low quality evidence and must be considered as “good practice”-guidelines.

Volar plating of DRF may harm patients. Complication rates of up to 33% have been reported in surgically treated DRF patients [10–14]. Our own estimation of the complication rate after volar plating of DRF is 14.6% [95% CI 11.8–17.7%] in a retrospective cohort of 595 patients with 3.2 years follow-up [3]. This high complication rate is not insignificant for the patients. Neurologic disturbances, tendon irritation and rupture, infection, etc. often lead either to a re-operation or an increase in out-patient visits and may result in permanent morbidity and impaired function [15].

Furthermore, volar plating improves the early functional recovery, but long-term functional results are similar with other treatment modalities in patients ≥65 years [16]. However, volar plating is still the gold standard in the treatment of DRF in adults regardless of age.

An increase in complications with no clinically significant difference between the functional outcome of operatively and conservatively treated patients was demonstrated [16, 17]. Furthermore, the patients treated operatively had a higher complications rate than the conservatively treated patients [17]. In addition, patients with DRF treated nonoperatively have shown to have less pain and better or equal wrist function after a 1 year follow-up than those treated surgically [18]. A Danish review of operatively treated patients suggest a more restrictive choice of treatment for DRF amongst the elderly than the NCG stipulate [2]. All things considered, no existing evidence proves the benefit of treating DRF operatively in the elderly.

Here, we question whether the potential benefit of volar plating, namely an earlier functional recovery outweighs the risk of encountering a complication. Especially in the light of retired patients (≥ 65 years), where return to work is not a burning issue, it seems worthwhile to investigate this issue both in the interest of the patients and society.

### Research hypothesis

Patients above 65 years of age, who sustain a DRF that fulfill the national radiologic criteria for operative treatment will experience fewer complications when treated with dorsal plaster cast immobilization only than when operated using a volar locking plate.

Meanwhile, the secondary outcome measures will be comparable and below the clinically relevant difference – e.g. a mean difference in the Danish version of the Quick Disabilities of the Arm, Shoulder and Hand (Quick DASH) below 16–20 point [19–22].

## Methods/design

### Study design

**Fig. 1:**
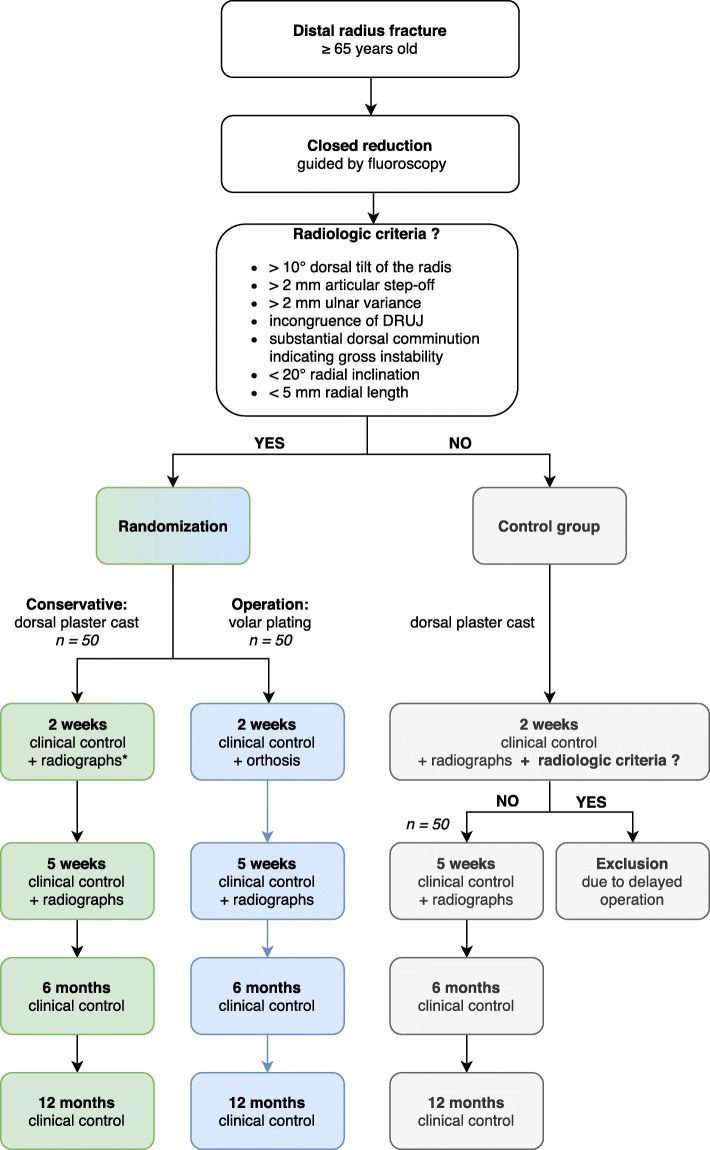
Study Design. An overview of the study design showing the 3 arms. *These radiographs are evaluated respectively after study completion and do not influent the prospective follow-up of the individual patient

A prospective single-center, single-blinded randomized-controlled superiority trial with two parallel treatment arms and a third control arm (Fig. 1);

- Arm 1: volar plating, 2-weeks dorsal plaster cast followed by 3-weeks orthosis immobilization with a single hand therapeutic instruction;
- Arm 2: closed reduction and 5-weeks dorsal plaster cast immobilization with a single hand therapeutic instruction;
- Arm 3: a control group of patients with minimally displaced DRF that do not fulfill the radiologic criteria.

Follow-up time is planned to be 1 year with out-patient visits at 2 weeks, 5 weeks, 6 months and 12 months after the injury. This trial is approved by the Danish Scientific Ethical Committee on the 3rd of September 2018. This study is carried out at the Regional Hospital Randers, Denmark with a coverage area of approximately 270,000 inhabitants.

### Eligibility criteria

All patients with DRF diagnosed at the emergency department are screened for eligibility.

#### Intervention groups: (arm 1 and arm 2)

Eligibility criteria for participants who will be allocated to random treatment are:

- ≥ 65 years old
- low-energy distal radius fracture.

The distal radius fracture must fulfill at least *one* of the following radiological criteria after closed reduction in the emergency department in order to be randomized between treatment arm 1 and 2:

- > 10° dorsal tilt of the radius in relation to perpendicular to the longitudinal axis of radius
- > 2 mm Ulnar variance
- > 2 mm Articular step-off
- Incongruence of the distal radioulnar joint
- Substantial dorsal comminution
- < 20° Radial inclination
- < 5 mm Radial length

#### Control group: (arm 3)

Eligibility criteria for participants in the control group, arm 3:

- ≥ 65 years old
- low-energy distal radius fracture.

This distal radius fracture had to fulfill *all* the following radiologic criteria:

- ≤ 10° Dorsal tilt of the radius in relation to perpendicular to the longitudinal axis of radius
- ≤ 2 mm Ulnar variance
- ≤ 2 mm Articular step-off
- No incongruence of the distal radioulnar joint
- ≥ 20° Radial inclination
- ≥ 5 mm Radial length

### Exclusion criteria

- Patients < 65 years
- high-energy fracture
- open fracture
- concomitant injuries, e.g. multiple fractures on afflicted arm
- not capable of giving written consent
- previous DRF or forearm fracture on the same side

### Recruitment

Any participant must be approved eligible for the study by either one of the consultants in the research group or the house physician on call. Patients are primarily recruited by directly contact in the emergency room on the day of primary contact, where they are informed about the study and asked for written consent. The Danish consent form and patient information material is given to the patient, a blank sample can be ordered from the corresponding author. Every patient who is treated in the emergency department during a shift is discussed the following day on a conference, where all radiographs also are reviewed. This additional control ensures that every potential participant is assed for eligibility and offered enrollment in the study either directly in the emergency room or the day after by telephone. When recruitment is done over the telephone, written consent is obtained before surgery or at the 2 week out-patient visit, if the participant should be randomly assigned to conservative treatment or if the patient is in the control group. The recruiting health care personnel randomly assigns participants to the interventions as described below.

### Randomization

**Fig. 2:**
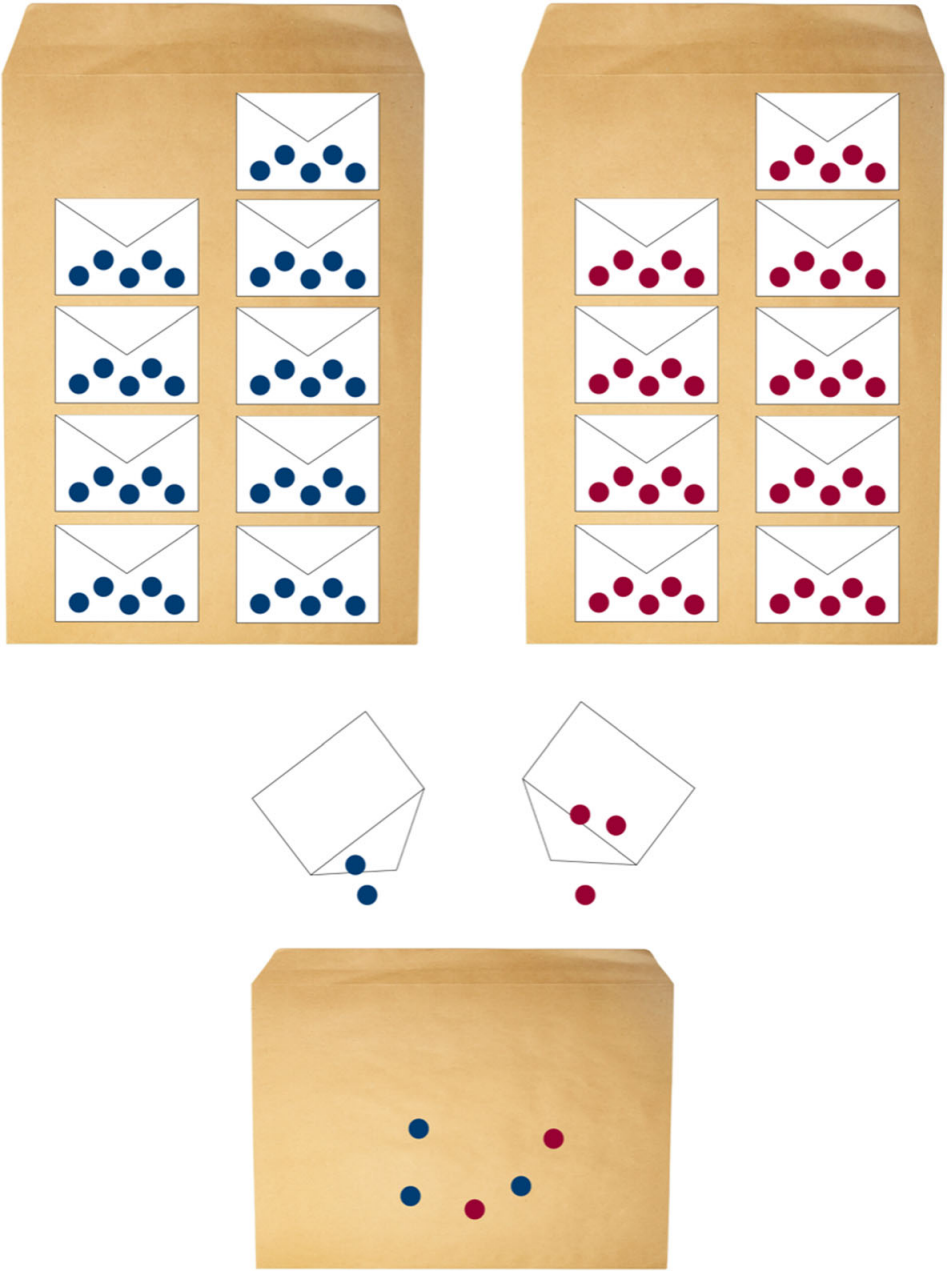
Illustration of the randomisation process regarding intervention groups, e.g. treatment arm 1 and 2. Each dot represents a sealed, opaque envelope that contains a note with the treatment arm allocation. The blue and red dots represent “conservative” and “operative” treatment, respectively. Whenever only 3 envelopes are left, 5 new “operative” and 5 new “conservative” treatment envelopes are mixed and added

Randomization is executed by random drawing of sealed, opaque envelopes. According to the sample size calculation, 50 participants will be allocated to each group, hence 100 identical A5 envelopes have been sealed – each containing a folded note whereupon either “*operative”* or “*conservative”* is written. In order to assure similar timewise enrolment the following measures will be applied (Fig. 2). The 50 envelopes for operative treatment will be packed into stacks of 5 envelopes. The same will be done with the 50 envelopes for conservative treatment. One stack of each treatment arm will be mixed resulting in an equal chance to draw either treatment or intervention among the ten envelopes. The including health care personnel will draw one of the 10 envelopes and hence allocate the participant randomly to either treatment arm 1 or 2. When there are only three envelopes left, one stack of each group will be opened and mixed into the remaining. By this measure, the health care personnel cannot predict the allocated treatment based upon the order of previous mixed treatment allocation from the mixed pool of small envelopes.

### Interventions

#### Interventions when a possible participant addresses the emergency room

After diagnosing the fracture on a standardized wrist radiograph (anterior-posterior projection and lateral projection) in the emergency room, the physician on call has two attempts to achieve an acceptable closed reduction under local analgesia with a 20 mg/ml Lidocaine hematoma block. While the effect of the hematoma block sets in, nurses measure the arm to be able to lay a proper dorsal plaster cast immobilization. Fluoroscopy is readily available in the emergency room and guides the closed reduction and plaster immobilization. After reduction standardized radiographs are obtained at the department of radiology and the quality of the closed reduction is assessed by the physician on duty. If the radiologic eligibility criteria are fulfilled after closed reduction, the patient is informed about the study and offered enrollment. If the fracture is less severe, e.g. without any of the radiologic criteria warranting closed reduction or operation as mentioned above, the wrist is immobilized with a plaster cast without closed reduction.

#### Intervention group

Treatment arm 1:

Open reduction and volar plate fixation utilizing Acu-Loc®, Acumed or Variax®, Stryker with a standard Henry approach to the distal radius and pronator quadratus repair if possible. The vast majority of patients will be operated in regional anesthesia and the remaining patients in general anesthesia. It is the choice of the surgeon whether a tourniquet will be used. After surgery the wrist is immobilized in a dorsal plaster cast for 2 weeks followed by further 3 weeks of immobilization with a removable orthosis. A single hand therapeutic instruction will take place.

Treatment arm 2:

Conservative treatment consists of dorsal plaster cast immobilization for 5 weeks. Only discomfort, neurologic deficits or signs of infection warrant removal and replacement with another dorsal plaster cast. A single hand therapeutic instruction will take place.

#### Control group

Patients with less displaced fractures, before or after and eventual closed reduction will be:

- Conservatively treated as described for treatment arm 2.
- Patients in the control group that fulfill the radiologic criteria after 2 weeks due to loss of reduction, are offered operative treatment according to the NCG and hence will be excluded from the trial.
- The investigators reserve the right to exclude a participant if it is considered clinically irresponsible to let them continue.

### Outcomes

Summarized in Table 1.

**Table 1:** Illustration of timeline and outcome measures including baseline demographics

	DRF	2 weeks	5 weeks	6 months	12 months
Primary outcome complications					
Questionaire	x	x	x	x	x
Examination	x	x	x	x	x
Secondary outcome					
Patient reported outcome					
Quick DASH (DK)	x	x	x	x	x
PRWHE (DK)				x	x
EQ5D				x	x
Pain at rest (0–10)	x	x	x	x	x
Objective examination					
Wrist range of motion			x	x	x
Grip strength				x	x
Pinch gauge				x	x
Wrist radiographs	x	x	x		
Baseline demographics:					
Age, gender, hand dominance, working status, ASA class, diabetes, smoking, alcohol consumption					

Illustration of timeline and outcome measures including baseline demographics Abbreviations: *Quick DASH (DK)* The quick disability of the arm, shoulder and hand outcome measure - validated Danish translation, *PRWHE (DK)* Patient-related wrist evaluation score - validated Danish translation, *ASA class* American Society of Anesthesiologists Classification

#### Primary outcomes

The complication rate will be estimated at day 0, 2 weeks, 5 weeks, 6 months, and 12 months after the injury.

Complications are defined as the presence of:

- Sensory disturbance, including carpal tunnel syndrome and chronic regional pain syndrome
- Flexor tendon rupture and irritation
- Extensor tendon rupture and irritation
- Hardware failure, e.g. osteosynthesis loosening
- Infection: superficial
- Infection: deep
- Reoperation with hardware replacement
- Reoperation with hardware removal (partial or total), which is not routinely performed in Denmark
- Vascular compromised (capillary refill ≥2 s)

Patients will report complications at the given timepoints by answering a questionnaire stating either yes / no and a free-text explanation. If the patient states any complications, a member of the research group will qualify the answer and fill in the free text. However, a YES can only be qualified and shall never be erased if the physician does not agree with the patient’s opinion or explanation.

The Danish version of the Quick Disabilities of the Arm, Shoulder and Hand [22] will be used to assess the level of functionality prior to injury, after 2 weeks, 5 weeks, 6 months, and 12 months. The minimal clinically relevant difference is 16 to 20-point difference in Quick DASH [19–21].

**Fig. 3:**
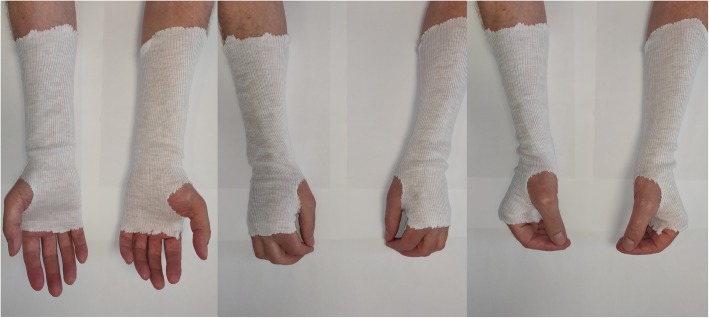
A demonstration of the blinding of treatment using a glove

Range of motion is measured by a registered nurse using a goniometer. To ensure the observer is blinded, the patient is instructed not to talk about the treatment. Furthermore, all wrists will be covered by a glove concealing potential scares (Fig. 3). The following data are thus collected in a blinded fashion wrist flexion, extension, pronation, supination, radial deviation, ulnar deviation. The contralateral side will serve as a reference and history of injuries or operations of the contralateral side will be recorded.

#### Secondary outcomes

The patient reported outcome measure Quick DASH will be supported by the following secondary outcome measures:

- ▪ A Danish version of the Patient rated Wrist Evaluation questionnaire (PRWE) will be evaluated after 6 month and 12 months [23].
- ▪ Grip strength of both left and right hand will be estimated as the maximum and average of score of three repetitions of each hand with alternating hands between attempts after 6 months and 12 months using a dynamometer.
- ▪ A pinch gauge where both left and right hand are evaluated (yes/no) if the participant can pinch a sheet of paper. This is collected after 6 months and 12 months by an unblinded physician or research year student.
- ▪ The potential flexion deficit of the 1st finger towards the base of the 5th finger measured as the distance (cm) from the pulp of the 1st finger to the carpometacarpal joint of the 5th finger after 6 and 12 months.
- ▪ The pulp-to-palm distance of the distal 2nd-5th finger and palmar surface of the side treated for DRF after 6 and 12 months.
- ▪ The experienced pain during activity within the preceding 14 days before the injury and at 6- and 12-months follow-up stated on a 0–10 Numeric Rating Scale (NRS).
- ▪ The pain at rest after 2 and 5 weeks given NRS.
- ▪ EQ5D (European Quality of life – 5 Dimensions) after 6 months and 12 months. This is registered by an unblinded physician or research year student [24].
- ▪ The self-reported use of pain medication at day 0, after 2 weeks, 5 weeks, 6 months, and 12 months.
- ▪ The prescribed use of pain medication compared 12 months before with 3 and 12 months after the injury.
- ▪ Standardized radiographs of the injured wrist at day 0 before and after closed reduction (all groups), week 2 (conservative treatment group, not reviewed before completion of the follow-up period), week 5 (all groups).

*The following baseline demographics* will be recorded: gender, age, side of DRF, hand dominance (right-handed, left-handed, ambidextrous), working status, American Society of Anesthesiologists Classification (ASA class 1–6), smoking (cigarettes/day), alcohol consumption (units/week) and diabetes (yes/no).

### Blinding

The study is single blinded, as all measurements will be performed with a glove masking a potential scare. Hence, the observer will be blinded when examining the participant. Furthermore, before each visit the patient is instructed, not to talk about the received treatment with the observer.

During the planning of the study, sham-operations were taken in consideration in order to blind the participants. However, most DRF are operated wide-awake under regional anesthesia only. The research group considered performing a skin incision and writing a manuscript simulating all the noises and communication, that usually take place during an operation. However, the efficacy of this potential blinding of participants during wide-awake surgery was deemed questionable. General anesthesia would have been a viable option, but it would vary too much from the current practice to be feasible at our hospital.

### Surgeon experience and type of plates

Our previous retrospective follow-up study concluded, that neither surgeon experience nor type of volar locking plate was associated with the complication rate [3]. Therefore, we consider surgeon experience and type of plate of no to minor clinical importance for the outcomes of this study. Hence, all physicians that usually treat DRF conservatively and operatively at our hospital will treat patients. No selection, nor restrictions regarding treating physician and fracture type will be imposed and operating physicians range from residents to consultants.

### Statistical plan and analysis

#### Sample size

The sample size was calculated based on a 20% difference in complication rate between the two treatment groups, an alpha level of 5% and a power of 80%. Consequently, each group shall at least consist of 49 participants. The control group was decided to be of equal size.

#### Data management

All data will be managed in accordance with Good Clinical Practice. Papers containing patient identifiable data along with informed consent are physically stored in a locked room. Study data will be collected and managed using REDCap electronic data capture tools hosted at Aarhus University, Denmark [25]. The Data Steering Committee (RT, JDR, JP) will review included and excluded patients every 14th day. If patients do not show up for follow-up in the outpatient clinic, The Data Steering Committee will contact the patient by phone and/or mail in order to ensure participant retention and complete follow-up. Only the Data Steering Committee will have access to the final trial data set. No publication of the data is planned; however data will be stored according to national legislation.

#### Statistical analysis plan

That data will be analyzed using Fisher’s exact test and Mann Whitney U test. The desired applied statistic is odds ratio with Pearson’s 95% confidence interval. Should any data be lost in the follow-up, the Last Observation Carried Forward concept will be used. Treatment arm 3 will be analyzed after 6 months follow-up and published separately.

All test will be two-tailed and assessed at the 5% alpha level. Categorical measures will be presented as percentages. An intention-to treat and per-protocol analysis will be considered. Continuous measures will be presented as means with standard deviations and medians with inter-quartile range. Treatment effects over time will be assessed using linear mixed effect models with patient treated as random factor. A normal distribution with an identity link function will be assumed for continuous measures, while a multinomial distribution and cumulative logit function will be applied to ordinal outcomes.

## Discussion

This prospective trial helps to clarify the best treatment strategy for displaced DRF patients ≥65 years. Practical limitations prevent the conduction of a double-blinded data collection, as no sham operations will be performed.

To the best of our knowledge, only one similar randomized controlled trial investigating volar locking plates versus conservative treatment in patients ≥65 years with DRF has been conducted [8, 16]. In 2011, Arora and co-workers reported similar results in terms of patient-reported outcome measures DASH and PRWE, pain level and range of motion between 36 operatively and 37 non-operatively treated patients [16], while the complication rate was 36 and 14% in the two groups, respectively. However, this trial did not have a significant clinical impact on the treatment of this patient group in Denmark. In the light of these promising initial report limiting the need of surgical intervention, there is a need to verify its results. Thus, the current trial will help to clarify the best treatment strategy for displaced DRF in patients ≥65 years in terms of complication rate and expected comparable functional outcome. If the results of the study indicate one treatment superior to the other, clinical guidelines are likely to be influenced by the current study.

Furthermore, cost effectiveness calculations can be performed on the basis of results of the current study and unnecessary operations may be prevented in order to live up to the Hippocratic oath, ‘primum non nocere - first do no harm’.

## Data Availability

Not applicable.

## Abbreviations

ASA class: American Society of Anesthesiologists Classification
DRF: Distal radius fractures
EQ5D: European Quality of life – 5 dimensions
NCG: National Clinical Guidelines
NRS: Numeric Rating Scale
ORIF: Open reduction internal fixation
PRWE: Patient rated Wrist evaluation
Quick DASH: Quick Disabilities of the Arm, Shoulder and Hand
